# Over- and Under-sampling Approach for Extremely Imbalanced and Small Minority Data Problem in Health Record Analysis

**DOI:** 10.3389/fpubh.2020.00178

**Published:** 2020-05-19

**Authors:** Koichi Fujiwara, Yukun Huang, Kentaro Hori, Kenichi Nishioji, Masao Kobayashi, Mai Kamaguchi, Manabu Kano

**Affiliations:** ^1^Department of Material Process Engineering, Nagoya University, Nagoya, Japan; ^2^Department of Systems Science, Kyoto University, Kyoto, Japan; ^3^Health Care Division, Japanese Red Cross Kyoto Daini Hospital, Kyoto, Japan

**Keywords:** health record analysis, imbalanced data problem, boosting, over- and under-sampling, stomach cancer detection

## Abstract

A considerable amount of health record (HR) data has been stored due to recent advances in the digitalization of medical systems. However, it is not always easy to analyze HR data, particularly when the number of persons with a target disease is too small in comparison with the population. This situation is called the imbalanced data problem. Over-sampling and under-sampling are two approaches for redressing an imbalance between minority and majority examples, which can be combined into ensemble algorithms. However, these approaches do not function when the absolute number of minority examples is small, which is called the extremely imbalanced and small minority (EISM) data problem. The present work proposes a new algorithm called boosting combined with heuristic under-sampling and distribution-based sampling (HUSDOS-Boost) to solve the EISM data problem. To make an artificially balanced dataset from the original imbalanced datasets, HUSDOS-Boost uses both under-sampling and over-sampling to eliminate redundant majority examples based on prior boosting results and to generate artificial minority examples by following the minority class distribution. The performance and characteristics of HUSDOS-Boost were evaluated through application to eight imbalanced datasets. In addition, the algorithm was applied to original clinical HR data to detect patients with stomach cancer. These results showed that HUSDOS-Boost outperformed current imbalanced data handling methods, particularly when the data are EISM. Thus, the proposed HUSDOS-Boost is a useful methodology of HR data analysis.

## 1. Introduction

Digitalization of medical information is rapidly expanding due to advances in information technologies, and many governments and medical institutions worldwide are promoting the adoption of electronic health record (EHR) systems. An EHR system is a container for storing the collection of patient and population health information in a digital format and for sharing them over networks (1–3). A health record (HR) includes a wide range of items, such as patient demographics, medical history, medical images, prescription, laboratory test results, vital signs, and billing. According to the U.S. Department of Health and Human Services, more than 80 percent of hospitals in the U.S. had adopted EHR systems by 2014 (4). In Japan, 77.5% of 400-bed hospitals had introduced EHR systems by 2016, according to a survey by the Ministry of Health, Labour and Welfare (MHLW).

The use of EHR systems would improve the quality and efficiency of medical care, for example, by facilitating smooth transition of patients between hospitals, preventing unnecessary treatments and tests, and optimizing medical resources (5). Analysis of a significant amount of HR data will contribute to improving clinical decision-making, discovering hidden relationships between diseases and patient lifestyles, and predicting clinical endpoints (3).

It is beneficial to detect signs of a disease in its early stages without special examinations. From the viewpoint of machine learning, rare disease detection is formulated as a binary classification problem: persons with or without the disease. However, the majority of people will not contract a disease unless the target disease becomes prevalent, such as the cold or the flu. In this case, the objective data become imbalanced because the number of patients with the target disease is small while that of others is large.

Examples observed from the target rare event are referred to as minority class examples, and examples from frequent events are called majority class examples. Coping with the imbalance between majority and minority classes is a challenging problem for standard machine learning algorithms since most of them are designed for balanced data (6, 7). These algorithms that optimize model parameters based on classification accuracy tend to ignore the minority class. Consider a dataset with 99 majority examples and one minority example. A typical algorithm may classify all examples into the majority class because a classification accuracy of 99% is achieved. An accuracy of 99% means a highly-accurate classifier for the balanced data problem; however, such a classifier is unsatisfactory, since the detection of minority examples is of crucial importance in most imbalanced data problems. Although some methodologies for coping with the imbalanced data problem have been proposed, they do not always function well, particularly when the absolute number of minority examples is too small. In this work, such a situation is defined as an extremely imbalanced and small minority (EISM) data problem. HR data analysis frequently faces the EISM data problem.

The present work proposes a new boosting-based algorithm that combines heuristic under-sampling (HUS) and distribution-based sampling (DOS) to overcome the binary classification problem of EISM data, particularly for HR data analysis. The proposed method is referred to as boosting combined with HUS and distribution-based sampling (HUSDOS-Boost). HUS selects majority examples that may be important for subsequent weak classifier learning based on the former boosting results, and DOS generates multiple artificial minority examples whose variables are generated randomly in accordance with the distribution of the minority class. Through using these two sampling methods simultaneously, an artificially balanced training dataset is generated for weak classifier learning. In HUSDOS-Boost, multiple weak classifiers are constructed using classifications and regression trees (CARTs) (8). Finally, they are combined into a strong classifier for binary classification using the boosting method.

This paper is organized as follows: section 2 provides an overview of conventional algorithms for handling the imbalanced data problem. To cope with the EISM problem, HUSDOS-Boost is proposed in section 3. Section 4 evaluates the performance of the proposed HUSDOS-Boost through application to eight imbalanced datasets and discusses its characteristics. Section 5 reports the result of applying the proposed method to original clinical HR data. The objective here is to detect patients with stomach cancer from the HR data. Also, this section discusses variables relevant to stomach cancer development derived from the variable importance. Conclusion and future works are presented in section 6.

## 2. Related Works

Various methodologies for coping with the imbalanced data problem have been investigated because the imbalanced data problem is not limited to the medical field (9), and many real-world issues involve learning from imbalanced data, such as fraud detection (10) and oil spill detection (11). The imbalanced data problem arises due to characteristics of severe events like natural disasters. This phenomenon is sometimes called the power law (12).

This section explains existing methodologies for dealing with the imbalanced data problem, which are classified into six approaches–anomaly detection approach, cost-sensitive approach, rule-based approach, sampling approach, ensemble learning approach, and hybrid approach, which is a combination of the sampling approach and the ensemble learning approach.

### 2.1. Anomaly Detection Approach

One approach to deal with the imbalanced data problem is formulated as anomaly detection, which is also called one-class learning. One class support vector machine (OCSVM) and local outlier factor (LOF) are well-known anomaly detection algorithms (13, 14). Fujiwara et al. (15) used multivariate statistical process control (MSPC) for epileptic seizure prediction, which is a well-known anomaly detection method originally used in process control (16, 17). When interested in the discovery of hidden factors related to disease development from HR data, the importance of each variable to the outcome should be calculated. Such importance is not always calculated in an anomaly detection approach, although some methods have been proposed (18, 19).

### 2.2. Cost-Sensitive Approach

The main concept of cost-sensitive approaches is to introduce different miss-classification costs for different classes. For instance, if an algorithm incorrectly classifies a healthy person as a patient in a health check, the impact of misdiagnosis is not crucial. In contrast, a patient may lose an opportunity for treatment if he/she is diagnosed as healthy. In this example, the misclassification cost of the latter case is much higher than that of the former case. In general, the misclassification cost of the minority examples must be higher than that of the majority examples (20). Cost-sensitive support vector machine (C-SVM) is a well-known cost-sensitive algorithm, which introduces different costs for different classes into the support vector machine (SVM) (21).

### 2.3. Rule-Based Approach

Rule-based approaches find classification rules from the dataset. A major methodology of the rule-based approach is a decision tree. In the decision tree, a measure is needed to find the classification rules, of which information gain is widely used (22, 23). Some measures have been proposed in order to cope with the imbalance data problem. Liu et al. (24) proposed a class confidence proportion (CCP) measure which uses Fisher's exact test to prune branches that are not statistically significant. In addition, the rule-based approach can be combined with another machine learning method. Batuwita and Palade (25) proposed fuzzy-ruled SVM (FSVM) with the cost-sensitive approach, referred to as FSVM-CIL (FSVM with class imbalance learning), which copes well with the imbalanced data problem particularly when the data contains outliers.

### 2.4. Sampling Approach

The imbalanced numbers of examples between the majority class and the minority class are modified through sampling methods (9). Under-sampling deletes majority examples from the dataset so that the numbers of examples between different classes become balanced, of which random under-sampling (RUS) is a well-known method (26). Since under-sampling shrinks the data size, less time is necessary for learning. The disadvantage is that discarding majority examples may lead to losing useful information of the majority class.

Over-sampling is carried out to add minority examples to the dataset in order to achieve a balance, in which the existing minority examples are replicated, or artificial minority examples are generated. Random over-sampling (ROS) replicates the existing minority examples randomly and adds them to the dataset. However, it may cause overfitting because learning algorithms tend to focus on replicated minority examples. To avoid overfitting, over-sampling methods which generate artificial minority examples are preferred. Synthetic minority over-sampling technique (SMOTE) is a commonly used over-sampling method that randomly selects minority examples and creates artificial minority examples via random interpolation between the selected examples and their nearest neighbors (27). Some modifications of SMOTE for enhancing its performance by modifying minority example selection have been proposed. For instance, adaptive synthetic sampling (ADASYN) adaptively changes the number of artificial minority examples following the density of majority examples around the original minority example (28).

### 2.5. Ensemble Learning Approach

In order to use ensemble algorithms, like boosting and bagging, it is necessary to construct multiple weak classifiers by means of any learning algorithm and to integrate them into a final strong classifier. Although ensemble algorithms were not originally designed for handling imbalanced data problems, they perform relatively well in many imbalanced data problems (29). Random forest (RF) and Adaptive Boosting (AdaBoost) are well-known methods of ensemble algorithms (30–32). Moreover, these methods can calculate the importance of variables (33), which may contribute to discovering hidden factors of disease development in HR data analysis.

### 2.6. Hybrid Approach

Sampling approaches can be combined with ensemble learning algorithms, such as boosting and bagging, because ensemble learning algorithms tend to outperform other machine learning algorithms when dealing with the imbalanced data problem (9). Such combinations are called hybrid algorithms. Under-sampling or over-sampling methods for balancing classes are used for weak classifier learning in boosting or bagging. RUSBoost is a well-known hybrid algorithm that combines RUS and boosting (26). A hybrid approach method adopting a sampling method and hyper ensemble learning, which is referred to as hyperSMURF, has been proposed (34). Hyper ensemble learning is an meta-ensemble learning framework that combines classification results of multiple ensemble learning classifiers.

However, hybrid algorithms do not always function well, particularly when the objective data is EISM.

## 3. HUSDOS-Boost

The present work proposes a new method for coping with the imbalanced data problem, in particular, with the EISM data problem. The proposed HUSDOS-Boost combines HUS and distribution-based over-sampling (DOS) with the AdaBoost framework.

To deal with the EISM problem, such as detecting rare diseases from HR data, both under-sampling and over-sampling can be used. Although a large number of minority examples need to be generated by over-sampling, such manipulation may lead to overfitting because many similar minority examples exist in the dataset. To avoid overfitting, under-sampling, which reduces the number of majority examples, should be used in addition to over-sampling so that a class balance is achieved with the generation of a small number of artificial minority examples.

Let *S* = {(***x***_*n*_, *y*_*n*_)}(*n* = 1, ⋯ , *N*) be the dataset and ***x***_*n*_ and *y*_*n*_ = {−1, 1} denote variables and class labels, respectively. In the imbalanced data, $S^{\;maj}=\left\{\left(x_{n},y_{n}\right)|y_{n}=1\right\}$ and $S^{\;min}=\left\{\left(x_{n},y_{n}\right)|y_{n}=-1\right\}$ are the majority and the minority datasets, respectively, and *S* = *S*^*maj*^∪*S*^*min*^. *N*^*maj*^ = |*S*^*maj*^|.

### 3.1. AdaBoost

Although there are some variations in the algorithms in the AdaBoost framework, AdaBoost.M1 is described here. The present work aims to detect a specific disease from HR data, which is formulated as a binary classification problem. In this case, AdaBoost.M1 and AdaBoost.M2 result in the same algorithm, and the former is simpler than the latter (35).

A procedure of AdaBoost.M1 is described in Algorithm 1. In step 1, the boosting weights of each example, *D*_1,*n*_(*n* = 1, ⋯ , *N*), are initialized to 1/*N*. After initialization, weak classifier learning is repeated in steps 2–8. Step 3 trains the *t*th weak classifier *w*_*t*_ so that the following objective function *J*_*t*_ is minimized:

(1) $$\begin{array}{l} J_{t}=\overset{N}{\underset{n=1}{\sum }}D_{t,n}I\left(h_{t,n}\neq y_{n}\right) \end{array}$$

where *I*(*h*_*n,t*_ ≠ *y*_*n*_) is an indicator function which returns 1 if *h*_*n,t*_ ≠ *y*_*n*_ and 0 otherwise. The error ε_*t*_ is calculated in steps 4 and 5. Steps 6 and 7 update a parameter β_*t*_ and the boosting weights *D*_*t,n*_:

(2) $$\begin{array}{l} D_{t+1,n}=\frac{D_{t,n}}{Z_{t}}\times \{\begin{array}{ll} \beta_{t} & \text{if }h_{t,n}=y_{n}\\ 1 & \text{otherwise} \end{array} \end{array}$$

where *Z*_*t*_ is a normalization constant. After *T* iterations, the final classifier *H*(***x***) is built as a weighted vote of the *T* weak classifiers as follows:

(3) $$\begin{array}{l} H\left(x\right)=\underset{y\in Y}{\text{arg max}}\underset{\;t:h_{t}=y}{\sum }\log\left(1/\beta_{t}\right). \end{array}$$

**Algorithm 1:** AdaBoost.M1

1: Initialize the boosting weights *D*_*n*,1_ = 1/*N* for ***x***_*n*_ ∈ *S*.
2: **for** *t* = 1, …, *T* **do**
3: Train the *t*th weak classifier *f*_*t*_ so as to minimize *J*_*t*_.
4: Get estimate of ***x***_*n*_ ∈ *S*: *h*_*t,n*_ = *f*_*t*_(***x***_*n*_).
5: Calculate the error of *h*_*t,n*_, ε_*t*_: $\;\varepsilon_{t}=\sum_{n=1}^{N}D_{t,n}I\left(h_{t,n}\neq y_{n}\right)$
6: Set β_*t*_ = ε_*t*_/(1 − ε_*t*_).
7: Update the boosting weights *D*_*t*+1,*n*_ using Eq.(2).
8: **end for**
9: **return** The final classifier *H*(***x***).

### 3.2. Heuristic Under-sampling

Although random under-sampling (RUS) randomly extracts a part of the majority examples for weak classifier learning (26), the drawback is that it does not consider the contribution that each majority example makes to the classification.

The proposed HUS selects majority examples according to sampling weights $SW_{t,n}\left(t=1,\cdots T;n=1,\cdots \;,N^{maj}\right)$ which are updated based on the estimation results in each boosting iteration. The initial sampling weight *SW*_1,*n*_ for the majority examples $x_{m}\in S^{\;maj}$ is set to 1/*N*^*maj*^. After the *t*th boosting iteration, HUS updates the sampling weights *SW*_*t,n*_ based on the *t*th estimation result *h*_*t,n*_ = *w*_*t*_(***x***_*n*_) as follows:

(4) $$\begin{array}{l} SW_{t+1,n}=\frac{SW_{t,n}}{Z_{SW_{t}}}\\ \times \{\begin{array}{ll} \beta_{t} & \text{if }x_{n}\in {Ŝ}_{t}^{\;maj}\wedge h_{t,n}=y_{m}\\ 1/\beta_{t} & \text{if }x_{n}\in {Ŝ}_{t}^{\;maj}\wedge h_{t,n}\neq y_{m}\\ 1 & \text{if }x_{n}\in S^{\;maj}\wedge x_{n}\notin {Ŝ}_{t}^{\;maj} \end{array} \end{array}$$

where ${Ŝ}_{t}^{\;maj}$ is the *t*th learning set sampled from *S*^*maj*^, and *Z*_*SW*_*t*__ is a normalization constant.

This update rule means that the sampled and misclassified majority examples have a higher probability of being sampled in the subsequent training set ${Ŝ}_{t+1}^{\;maj}$, while the sampled and correctly classified examples have a lower probability of being sampled. That is, majority examples that may be important for improving classification performance tend to be sampled for the subsequent weak classifier learning. Note that the sampling weights *SW*_*t,n*_ are different from the boosting weights *D*_*t*+1, *n*_, although their update rules use the same parameter β_*t*_.

We refer to a method in which the random under-sampling in RUSBoost is replaced with HUS as HUSBoost.

### 3.3. Distribution-Based Over-sampling

Over-sampling methods that generate artificial minority examples increase the amount of information for weak classifier learning. This study proposes distribution-based over-sampling (DOS), which generates artificial values for the variables based on their distributions.

Categorical and continuous variables are considered here. Categorical variables are generated by following the proportion of each attribute in the minority class, *p*_*k*_ = *N*_*k*_/*N*_*a*_, where *N*_*a*_ and *N*_*k*_ are the number of examples in the minority class and the number of examples that have the attribute *k*, respectively. For example, it is assumed that the number of “male” is 15 and that of “female” is 9 in “gender,” and the generated values in “gender” have a probability of 15/24 of being “male” and 9/24 of being “female.”

Continuous variables are generated by following the continuous distribution estimated from the minority examples. When we assume that a variable “height” follows the Gaussian distribution *N*(μ, σ^2^), its mean μ and variance σ^2^ need to be estimated. Then, artificial values for 'height' are generated by following *N*(μ, σ^2^).

Correlated variables may be generated by chance in the process of over-sampling, and such samples may cause multicollinearity in multiple regression (36). The multicollinearity problem is a phenomenon in which the estimated regression coefficients in a multiple regression model greatly fluctuate in response to small changes in training data when there is correlation among input variables. The regression coefficients are estimated using the normal equation: ***b*** = (***X***^*T*^***X***)^1^***Xy***, where ***X*** is an input matrix and ***y*** is an output vector. The matrix (***X***^*T*^***X***) becomes ill-conditioned when there is correlation among input variables, which lead to unstable inverse matrix calculation (37). On the other hand, the learning process of CART does not contain the inverse matrix calculation. Thus, the proposed HUSDOS-Boost avoids the multicollinearity problem even if the correlated variables are generated by over-sampling.

### 3.4. HUSDOS-Boost

Algorithm 2 shows the proposed HUSDOS-Boost algorithm, which combines AdaBoost.M1 with both HUS and DOS. HUSDOS-Boost with AdaBoost.M1 can be easily modified to an algorithm using AdaBoost.M2.

**Algorithm 2:** HUSDOS-Boost with AdaBoost.M1

1: Initialize the boosting weights *D*_*n*,1_ = 1/*N* for ***x***_*n*_ ∈ *S*, and the sampling weights $SW_{1,n}=1/N^{maj}$ for $x_{n}\in S^{\;maj}$.
2: **for** *t* = 1, …, *T* **do**
3: Apply HUS with *SW*_*t,n*_ to *S*^*maj*^ to generate ${Ŝ}_{t}^{\;maj}$ with a size *N*_*u*_.
4: Apply DOS to *S*^*min*^ to generate ${Ŝ}_{t}^{\;min}$ with a size *N*_*o*_, where $S^{\;min}\subset {Ŝ}_{t}^{\;min}$.
5: ${Ŝ}_{t}={Ŝ}_{t}^{\;maj}\cup {Ŝ}_{t}^{\;min}$.
6: Train the *t*th weak classifier *f*_*t*_ from Ŝ_*t*_ so as to minimize Ĵ_*t*_.
7: Get hypothesis of ***x***_*n*_ ∈ *S*: *h*_*t,n*_ = *f*_*t*_(***x***_*n*_).
8: Calculate the error of *h*_*t,n*_, ε_*t*_: $\varepsilon_{t}=\sum_{\;n:h_{t,n}\neq y_{n}}D_{t,n}$.
9: Set β_*t*_ = ε_*t*_/(1 − ε_*t*_).
10: Update the boosting weights *D*_*t*+1,*n*_ by Eq.(2).
11: Update the sampling weights *SW*_*t*+1,*n*_ by Eq.(4).
12: **end for**
13: **return** The final hypothesis *H*(***x***).

In step 1, the boosting weights of each example *D*_1,*n*_ and the sampling weights of each majority example *SW*_1,*n*_ are initialized to 1/*N* and 1/*N*^*maj*^, respectively. After initialization, *T* weak classifiers are iteratively trained in steps 2–12. In step 3, HUS is applied to select *N*_*u*_ majority examples for the *t*th majority training set ${Ŝ}_{t}^{\;maj}$. On the other hand, DOS generates *N*_*o*_ artificial minority examples and adds them to *S*^*min*^ to construct the *t*th minority training set ${Ŝ}_{t}^{\;min}$ in step 4. The numbers of selected majority examples by HUS and added minority examples by DOS, *N*_*u*_ and *N*_*o*_, should be determined by considering the desired ratio of the majority examples to the minority examples. After the *t*th training set Ŝ_*t*_ is constructed, the *t*th weak classifier is trained in step 6. Note that the range of summation in the objective function is modified from Equation (1) in Algorithm 1:

(5) $$\begin{array}{l} {Ĵ}_{t}=\underset{n|y_{n}\in {Ŝ}_{t}}{\sum }D_{t,n}I\left(h_{t,n}\neq y_{n}\right). \end{array}$$

The *t*th error ε_*t*_ is calculated in steps 7–8. The following steps 9-11 update the parameter β_*t*_, the sampling weights *SW*_*t*+1, *n*_, and the boosting weights *D*_*t*+1, *n*_. After *T* iterations, the final hypothesis *H*(***x***) is built as Equation (3).

### 3.5. Classification and Regression Tree

Although any learning algorithm can be used for the weak classifier in the proposed HUSDOS-Boost, a classification and regression tree (CART) (8) is adopted in this work. In CART, variable importance can be obtained.

A CART model is a binary tree that is obtained by splitting a variable set into two variable subsets recursively so that the cost function for misclassification is minimized. In addition, some leaf nodes are pruned after tree construction to obtain simple tree structures. CART uses the Gini coefficient as the cost function, which is an indicator of uniformity of data distribution. The Gini coefficient of the *r*th node, *I*_*G*_(*r*), is defined as follows:

(6) $$\begin{array}{l} I_{G}\left(r\right)=1-\overset{K}{\underset{k=1}{\sum }}\left(\frac{n_{r}^{\left\{k\right\}}}{N_{r}}\right) \end{array}$$

where *N*_*r*_ and $n_{r}^{\left\{k\right\}}$ are the numbers of all examples and examples belonging to class *k*, respectively. *K* is the number of classes. The decrease in the Gini coefficient due to the splitting of the *r*th node, Δ*I*_*G*_(*r*), is expressed as

(7) $$\begin{array}{l} \Delta I_{G}\left(r\right)=I_{G}\left(r\right)-\underset{l=1,2}{\sum }w_{r_{l}}I_{G}\left(r_{l}\right). \end{array}$$

*I*_*G*_(*r*_*l*_)(*l* = 1, 2) are the Gini coefficients of the child nodes of the *r*th node. *w*_*r*_*l*__ is defined as *w*_*r*_*l*__ = *N*_*r*_*l*__/*N*_*r*_, where *N*_*r*_*l*__ denotes the number of examples in the *l*th child node. The split that gives the largest decrease should be searched. Thus, Δ*I*_*G*_(*r*) also indicates the variable importance for classification in CART (32).

Since a strong classifier is the weighted sum of multiple CART models in HUSDOS-Boost, the variable importance of the *p*th variable, VI_*p*_, is defined as the weighted sum of the decreases due to the *p*th variable splitting:

(8) $$\begin{array}{l} VI_{p}=\frac{1}{Z_{\text{VI}}}\underset{t}{\sum }\log\left(1/\beta_{t}\right)\Delta I_{G}^{t}\left(p\right) \end{array}$$

where $\Delta I_{G}^{t}\left(p\right)\left(t=1,\cdot ,T\right)$ is the Gini coefficient decrease due to the *p*th variable splitting in the *t*th CART model, and *Z*_VI_ is a normalization constant.

## 4. Case Study

This section investigates the performance and the characteristics of the proposed HUSDOS-Boost through its application to eight imbalanced datasets collected from the UCI Machine Learning repository (38). In this case study, random forest (RF), AdaBoost, SMOTE, ADASYN, RUSBoost, HUSBoost were tested for comparison.

### 4.1. Datasets

This case study used the following eight imbalanced datasets, which cover a wide variety of data sizes, imbalance ratios of the majority class to the minority class, and application domains.

- **Covertype**: Dataset for forest cover type estimation based on cartographic data, which consists of seven classes (27). “Ponderosa Pine” and “Cottonwood/Willow” were selected as the majority and minority classes.
- **Satimage**: Dataset for soil type classification from multi-spectral image data measured by a satellite (27). The smallest class “red soil” was the minority class, and other classes were considered the majority class.
- **Segment**: Dataset for object type prediction from outdoor image segmentation data (26). There are five classes, and the number of examples in each class is the same. “brick face” was selected as the minority class, and the rest was considered the majority class.
- **Pageblocks**: Dataset for block type classification of a document page layout, which consists of five classes. “graphic” with 115 examples was selected as the minority class, and the rest was considered the majority class.
- ***E. coli***: Dataset for protein localization site prediction consisting of eight classes. “Inner membrane without signal sequence” was the minority class, and the others were considered the majority class (39).
- **CTG**: Dataset of fetal heart rate (FHR) prediction from cardiotocography. There are ten types of FHR, and “type 3,” whose size is the smallest, was selected as the minority class, and the rest were considered the majority class.
- **Abalone**: Dataset for abalone age estimation using physical measurements of an abalone. The ages of the abalones range from 1 to 29 in the dataset. The ages of 9 and 18 were selected as the majority and the minority classes, respectively (40).
- **Yeast**: Dataset for predicting cellular localization sites, which consists of ten classes (27). The class “VAC” with only 30 examples was chosen as the minority class, and others were considered the majority class.

Table 1 shows the characteristics of eight datasets, in which #Var, #Minority, and #Majority denote the numbers of input variables, minority examples, and majority examples in each dataset, respectively, and Ratio is their imbalance ratio: #Minority/(#Majority+#Minority). Note that datasets in Table 1 are sorted in descending order of #Minority.

**Table 1:** Dataset Characteristics.

**Dataset**	**#Var**	**#Minority**	**#Majority**	**Ratio [%]**
Covertype	54	2,747	35,754	7.13
Satimage	19	626	5,809	9.73
Segment	36	330	1,980	14.3
Pageblocks	10	115	5,358	2.10
*E. coli*	7	77	259	22.9
CTG	21	53	2,073	2.56
Abalone	8	42	689	5.75
Yeast	8	30	1,464	1.35

### 4.2. Experimental Procedure

The classification performances of RF, AdaBoost, SMOTE, ADASYN, RUSBoost, HUSBoost, hyperSMURF, and the proposed HUSDOS-Boost were evaluated using the imbalanced datasets described in section 4.1.

In SMOTE, the number of artificial minority examples generated by over-sampling was the same as the original number of majority examples for obtaining a perfectly balanced dataset, and a CART model was constructed. RUSBoost and HUSBoost sampled the same number of majority examples as that of minority examples by under-sapling. In the proposed HUSDOS-Boost, the number of artificial minority examples generated by DOS was the same as the original number of minority examples, and the number of sampled majority examples by HUS was twice that of the original minority examples. Thus, *N*_*u*_ = *N*_*o*_ = #Minority in steps 3–4 in Algorithm 2. The weak classifier used in RF, AdaBoost, RUSBoost, HUSBoost, and HUSDOS-Boost was a CART model, and the maximum number of their constructed weak classifiers was 100. hyperSMURF used RF for hyper ensemble learning.

Each dataset was randomly divided into ten subsets, of which nine were used for modeling while the remaining one was used for validation. Modeling and validation were repeated ten times so that all subsets became the validation dataset once. The above procedure was repeated ten times for precise performance evaluation.

The computer configuration used in this case study was as follows: CPU: Intel Core i7-9700K (3.60GHz × 8 cores), RAM: 32GB, OS: Windows 10 Pro (64 bit), and the R language was used.

### 4.3. Performance Metrics

In standard machine learning problems, the overall accuracy is a metric for performance evaluation: however, it is not appropriate in this case study because an accuracy of 99% is achieved when the imbalance ratio is 1:99 and a stupid classifier discriminates all of the examples as the majority class.

The geometric mean (G-mean) of the sensitivity and the specificity was used in this work:

(9) $$\begin{array}{l} G_{\text{mean}}=\sqrt{\text{sensitivity }\times \text{ specificity}}. \end{array}$$

The G-mean measures the classification performance of a classifier for minority class examples as well as majority class examples, simultaneously. A low value of the G-mean indicates that the classifier is highly biased toward one class and vice-versa. Thus, the G-mean is an appropriate metric for evaluating the imbalanced data problem.

In addition, an area under the curve (AUC) of a receiver operating characteristic (ROC) curve and the area under the precision-recall curve (AUPRC) were used for evaluating the averaged performances of classifiers.

The average CPU time per modeling calculation was measured for each method.

### 4.4. Results and Discussion

Table 2 shows the sensitivity, the specificity,the G-mean, AUC, and AUPRC of each method in eight imbalanced datasets. The bold fonts indicate the best scores in the seven algorithms.

**Table 2:** Performances of seven methods.

**Dataset**	**Metrics**	**RF**	**AdaBoost**	**SMOTE**	**ADASYN**	**RUSBoost**	**HUSBoost**	**hyperSMURF**	**HUSDOSBoost**
Cover type	Sensitivity	0.65±0.02	0.87±0.01	0.71±0.02	0.74±0.01	0.98±0.00	0.81±0.01	**0.99±0.00**	0.83±0.01
	Specificity	**1.00±0.00**	0.99±0.00	0.97±0.00	0.92±0.00	0.96±0.00	0.99±0.00	0.84±0.01	0.97±0.00
	G-mean	0.81±0.02	0.93±0.00	0.83±0.01	0.82±0.01	**0.97±0.00**	0.90±0.00	0.91±0.01	0.90±0.00
	AUC	0.99±0.00	**1.00±0.00**	0.87±0.00	0.92±0.01	0.99±0.00	0.99±0.00	0.98±0.00	0.98±0.00
	AUPRC	0.90±0.00	**0.96±0.00**	0.53±0.01	0.43±0.01	0.93±0.01	0.92±0.00	0.83±0.01	0.87±0.01
Satimage	Sensitivity	0.52±0.02	0.63±0.02	0.68±0.02	0.89±0.01	0.91±0.02	0.70±0.01	**0.94±0.01**	0.75±0.01
	Specificity	**0.99±0.00**	0.98±0.00	0.93±0.01	0.80±0.01	0.86±0.00	0.95±0.00	0.83±0.00	0.93±0.00
	G-mean	0.72±0.01	0.79±0.01	0.79±0.01	0.84±0.01	**0.89±0.01**	0.82±0.01	0.88±0.00	0.83±0.00
	AUC	**0.96±0.00**	0.78±0.01	0.87±0.01	0.85±0.01	0.55±0.12	0.95±0.00	**0.96±0.00**	0.94±0.00
	AUPRC	0.78±0.01	0.18±0.00	0.47±0.02	0.32±0.01	0.09±0.03	**0.75±0.01**	0.74±0.01	0.71±0.00
Segment	Sensitivity	**0.99±0.00**	**0.99±0.00**	0.96±0.01	0.91±0.01	**0.99±0.01**	**0.99±0.01**	**0.99±0.00**	**0.99±0.00**
	Specificity	**1.00±0.00**	**1.00±0.00**	0.99±0.00	0.99±0.00	0.99±0.00	1.00±0.00	0.99±0.00	0.99±0.00
	G-mean	**0.99±0.00**	**0.99±0.00**	0.98±0.01	0.94±0.01	**0.99±0.00**	**0.99±0.00**	**0.99±0.00**	**0.99±0.00**
	AUC	**1.00±0.00**	**1.00±0.00**	0.98±0.01	0.96±0.01	**1.00±0.00**	**1.00±0.00**	**1.00±0.00**	**1.00±0.00**
	AUPRC	**1.00±0.00**	**1.00±0.00**	0.93±0.03	0.89±0.01	**1.00±0.00**	**1.00±0.00**	**1.00±0.00**	**1.00±0.00**
Pageblocks	Sensitivity	0.65±0.03	0.65±0.06	0.71±0.06	0.87±0.02	**0.95±0.03**	0.90±0.02	0.90±0.02	0.90±0.02
	Specificity	**1.00±0.00**	**1.00±0.00**	0.99±0.00	0.92±0.01	0.94±0.01	0.97±0.00	0.97±0.00	0.97±0.00
	G-mean	0.81±0.02	0.80±0.04	0.84±0.03	0.89±0.01	**0.94±0.01**	0.93±0.01	0.93±0.01	0.93±0.01
	AUC	0.97±0.01	0.98±0.00	0.91±0.04	0.93±0.02	0.58±0.11	**0.99±0.00**	**0.99±0.00**	**0.99±0.00**
	AUPRC	0.80±0.01	0.76±0.02	0.55±0.07	0.26±0.04	0.03±0.01	0.75±0.02	**0.77±0.01**	0.74±0.03
Ecoil	Sensitivity	0.77±0.03	0.76±0.04	0.87±0.06	0.92±0.02	0.91±0.06	0.91±0.04	**0.97±0.00**	0.92±0.03
	Specificity	0.94±0.01	0.94±0.02	0.87±0.02	0.87±0.02	0.87±0.02	**0.89±0.01**	0.76±0.02	**0.89±0.01**
	G-mean	0.85±0.02	0.84±0.03	0.87±0.03	**0.90±0.01**	0.89±0.03	**0.90±0.02**	0.86±0.01	**0.90±0.02**
	AUC	0.95±0.01	0.95±0.01	0.93±0.03	0.92±0.01	0.95±0.02	0.95±0.01	0.95±0.01	**0.96±0.01**
	AUPRC	0.86±0.03	0.86±0.03	0.77±0.09	0.74±0.04	0.85±0.04	0.85±0.03	0.83±0.04	**0.87±0.03**
CTG	Sensitivity	0.55±0.05	0.67±0.07	0.65±0.08	0.79±0.05	0.93±0.07	0.89±0.02	0.87±0.04	**0.92±0.02**
	Specificity	**1.00±0.00**	1.00±0.00	0.98±0.01	0.96±0.00	0.91±0.01	0.96±0.00	0.98±0.00	0.96±0.00
	G-mean	0.74±0.03	0.82±0.04	0.80±0.05	0.87±0.03	0.92±0.03	0.93±0.01	0.92±0.02	**0.94±0.01**
	AUC	0.99±0.01	0.96±0.08	0.92±0.04	0.86±0.04	0.70±0.11	0.97±0.00	**0.98±0.00**	**0.98±0.01**
	AUPRC	0.78±0.02	0.65±0.55	0.45±0.06	0.42±0.08	0.09±0.04	0.72±0.04	0.68±0.03	0.73±0.03
Abalone	Sensitivity	0.15±0.05	0.37±0.05	0.46±0.10	0.60±0.09	**0.69±0.07**	0.57±0.02	0.76±0.03	0.67±0.10
	Specificity	**1.00±0.00**	0.99±0.01	0.92±0.02	0.83±0.01	0.74±0.03	0.87±0.02	0.80±0.01	0.86±0.01
	G-mean	0.38±0.07	0.61±0.04	0.65±0.07	0.71±0.05	0.72±0.04	0.70±0.02	**0.78±0.02**	0.76±0.05
	AUC	0.82±0.02	0.81±0.05	0.74±0.08	0.73±0.05	0.67±0.11	0.82±0.01	0.83±0.01	**0.84±0.03**
	AUPRC	0.44±0.05	0.44±0.08	0.27±0.11	0.24±0.08	0.19±0.08	0.37±0.06	0.40±0.07	**0.42±0.06**
Yeast	Sensitivity	0.00±0.00	0.03±0.04	0.07±0.03	0.23±0.10	0.60±0.10	0.43±0.10	0.31±0.08	**0.56±0.08**
	Specificity	1.00±0.00	1.00±0.00	0.98±0.01	0.85±0.02	0.57±0.03	0.84±0.01	**0.90±0.01**	0.74±0.01
	G-mean	0.00±0.00	0.14±0.14	0.25±0.07	0.44±0.10	0.58±0.04	0.60±0.07	0.53±0.07	**0.64±0.04**
	AUC	0.62±0.02	0.62±0.06	0.59±0.14	0.55±0.04	0.54±0.08	0.67±0.02	**0.70±0.02**	0.66±0.03
	AUPRC	0.05±0.03	0.08±0.05	0.06±0.04	0.03±0.01	0.03±0.03	**0.09±0.02**	0.05±0.01	0.06±0.04

RF and AdaBoost, which do not employ sampling methods, achieved high specificities while their sensitivities were lower than the three algorithms with sampling methods, which resulted in low G-means. SMOTE, which uses over-sampling and which are not an ensemble algorithm, performed modestly. ADASYN improved the performance of SMOTE, which showed that adaptive changes in the number of artificial minority examples is certainly effective. These results indicate that sampling method are effective in the imbalanced data problem.

RUSBoost, which uses random under-sampling and boosting, achieved the highest G-means in four datasets whose number of minority samples are the first to the fourth largest among the eight datasets. However, AUC and AUPRC of RUSBOOST achieved modest values, which means that its averaged performance is not so high. HUS-Boost that combines HUS and boosting kept rather high AUC and AUPRC when the imbalance ratio of a dataset was low although other performance metrics were modest. This indicated that HUS was effective when the imbalance ratio is low. hyperSMURF, which adopts hyper ensemble learning, achieved high performance on average even when the number of minority examples was rather small.

The proposed HUSDOS-Boost, which utilizes both over-sampling and under-sampling in addition to boosting, achieved the best G-means in five datasets whose numbers of minority samples are the third to the eighth largest. These results suggest that HUSDOS-Boost achieves higher performance than RUSBoost and HUSBoost when the imbalance ratio of a dataset is not particularly low, but the absolute number of minority examples contained in a dataset is minimal. In addition, HUSDOS-Boost also kept high AUC and AUPRC when the imbalance ratio was low, which means that its averaged performance does not deteriorate when the number of minority examples is minimal. Thus, the use of both HUS and distribution-based over-sampling is certainly effective.

**Figure 1:**
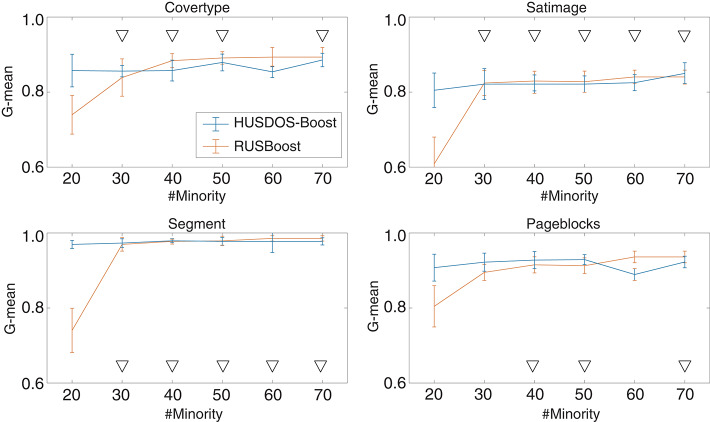
G-means of HUSDOS-Boost and RUSBoost vs. #Minority.

To verify this point, we compared RUSBoost and HUSDOS-Boost through another experiment using datasets with intentionally reduced minority examples. The minority examples in Covertype, Satimage, Segment, and Pageblocks, which have more than 100 minority examples, were eliminated randomly. The numbers of reduced minority examples in these datasets were 20, 30, 40, 50, 60, and 70. The procedure described in section 4.2 was applied to these reduced datasets. Figure 1 shows the G-means of RUSBoost and HUSDOS-Boost for the reduced datasets. The proposed HUSDOS-Boost performed better than RUSBoost when the number of minority examples was 20 and 30 regardless of #Var, and the performance of HUSDOS-Boost was almost the same as RUSBoost when the number of minority examples was more than 40. Thus, over-sampling, as well as under-sampling, should be used when the number of minority examples is small. It is concluded that the proposed HUSDOS-Boost is more appropriate than RUSBoost for solving the EISM data problem.

**Figure 2:**
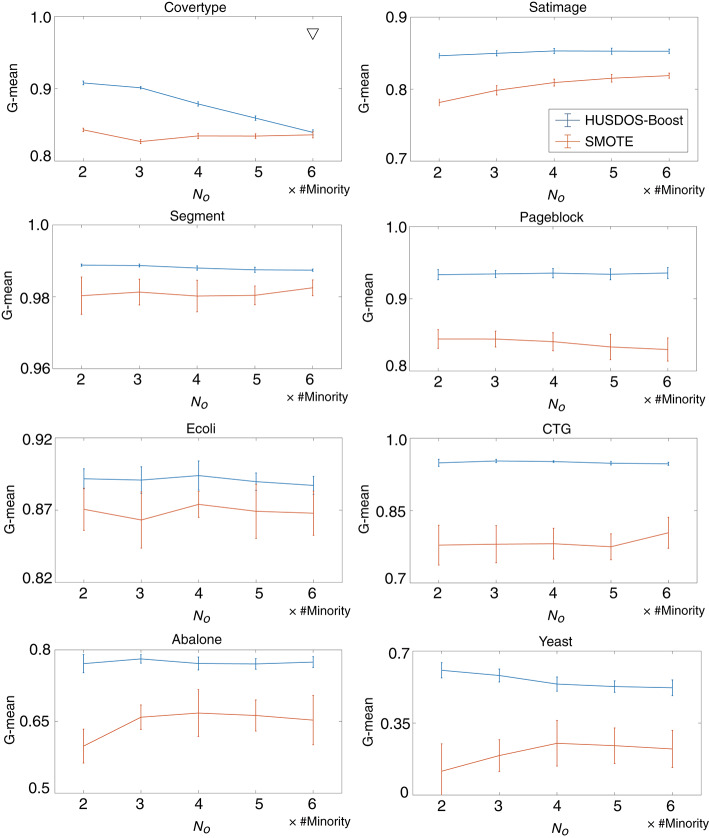
G-means of HUSDOS-Boost and SMOTE vs. *N*_*o*_.

To evaluate the effects of the number of examples generated by over-sampling *N*_*o*_, we investigated the performance of HUSDOS-Boost and SMOTE with *N*_*o*_ = {2, 3, 4, 5, 6}× #Minority using eight datasets. The number of examples sampled by under-sampling *N*_*u*_ is fixed to #Minority. Figure 2 illustrates the G-means of HUSDOS-Boost and SMOTE calculated for each *N*_*o*_. The ▽ marks in the figures denote the pairs for which a significant difference was not confirmed by the *t*-test (α = 0.05). These results show that the proposed HUSDOS-Boost achieved a higher performance than SMOTE regardless of which *N*_*o*_ was selected, and that the performance did not improve even when the number of artificial examples generated by over-sampling became large in most cases, which indicates that an excessive number of similar minority examples do not contribute to classifier learning.

**Figure 3:**
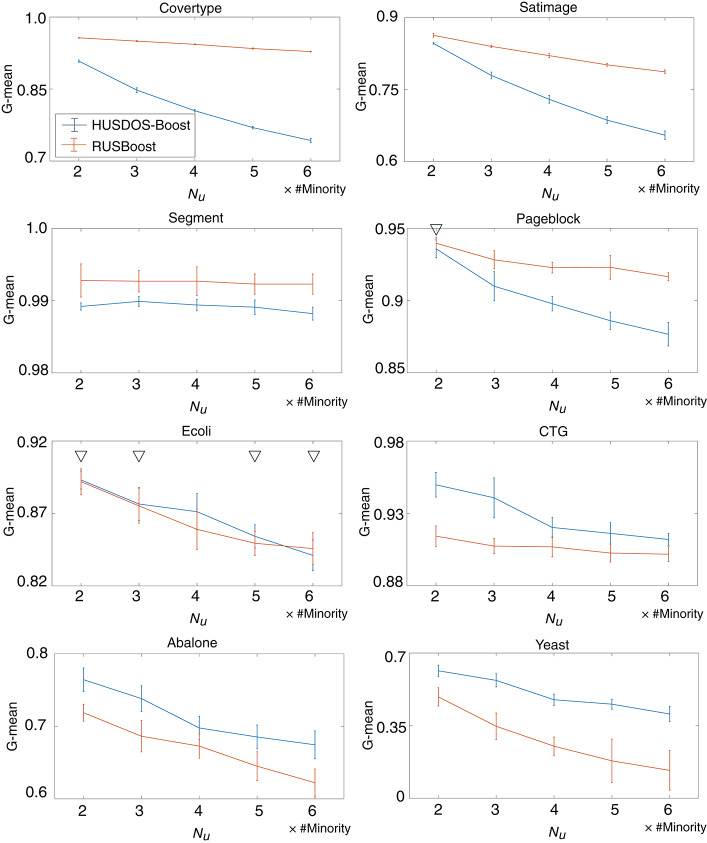
G-means of HUSDOS-Boost and RUSBoost vs. *N*_*u*_.

The influence of the number of majority examples sampled by under-sampling on classifier learning was checked. We tested HUSDOS-Boost and RUSBoost with *N*_*u*_ = {2, 3, 4, 5, 6}× #Minority using eight datasets and *N*_*o*_ = #Minority. Their G-means are illustrated in Figure 3, which shows that their performances deteriorated as *N*_*u*_ became large. Thus, the numbers of majority examples used for classifier learning should be balanced with the numbers of minority examples.

The average CPU times of each of the seven methods required for one strong classifier learning are reported in Table 3. In almost all datasets, RF was the fastest, in which multiple CARTs are constructed using a bagging approach in parallels. SMOTE was the second-fastest. Although SMOTE roughly doubled the number of examples for learning through over-sampling in this case study, just one CART model was built. Thus, the total amount of calculation was not significant. AdaBoost performed the worst because it uses all examples for weak classifier learning, and the learning process has to be performed in series. In hyperSMURF, the CPU times did not decrease so much when the number of examples became small because it constructed multiple RFs as hyper ensemble learning. The CPU times of RUSBoost were modest. Although RUSBoost is based on boosting in the same manner as AdaBoost, the number of examples used for weak classifier learning is significantly reduced due to under-sampling. Since RUSBoost was much faster than HUSBoost and the computational burdens of HUSBoost and HUSDOS-Boost were almost at the same level, heuristics under-sampling requires heavy computational burden although it is more effective than random under-sampling for the imbalanced data problem.

**Table 3:** CPU times (s).

	**RF**	**AdaBoost**	**SMOTE**	**ADASYN**	**RUSBoost**	**HUSBoost**	**hyperSMURF**	**HUSDOSBoost**
Cover type	**41.4±3.57**	4,635±1,582	119.0±13.9	66.4±10.1	285.6±18.1	3,978±90.6	402.7±14.8	3800±328
Satimage	**5.05±0.23**	321.0±12.0	6.08 ±0.88	11.4±0.46	130.6±9.34	191.7±2.99	47.89±1.22	206.1±3.96
Segment	**0.68±0.03**	122.3±3.11	1.09 ±0.18	0.76±0.02	101.5±6.69	39.9±1.71	38.9±0.93	44.7±1.16
Pageblocks	**1.15±0.18**	146.6±3.25	0.90 ±0.10	1.64±0.02	99.3±1.47	36.3±1.13	37.0±0.87	39.4±0.86
Ecoil	**0.09±0.02**	105.5±3.66	0.20 ±0.01	0.21±0.01	97.3±3.41	4.90±0.18	36.5±2.21	5.92±0.20
CTG	**0.68±0.06**	127.2±3.61	0.48 ±0.03	1.14±0.02	97.9±2.59	11.4±0.29	36.8±2.30	13.3±0.47
Abalone	**0.20±0.19**	108.8±2.77	0.21 ±0.01	0.32±0.01	97.0±4.41	6.00±0.20	36.5±1.97	7.14±0.34
Yeast	**0.32±0.03**	113.8±2.32	0.27 ±0.03	0.52±0.00	97.8±5.76	6.53±0.24	36.3±0.83	6.88±1.11

The variable importance is discussed in the following section 5.

## 5. Stomach Cancer Screening From Clinical Health Record Data

Early detection of stomach cancer is essential for its prognosis; however, stomach cancer detection is a typical EISM data problem. The lifetime morbidity risk of stomach cancer is 11% in males and 5% in females, and newly diagnosed patients per year is about 0.1–0.2% of the population in Japan. Hence, the number of patients with stomach cancer in the HR data is small, while those without stomach cancer is large. Although it is challenging to find stomach cancer at early stages due to lack of subjective symptoms, stomach cancer detection from HR data would be beneficial. The 5-year survival rate of stomach cancer is 82% for stage I while it is 8% for stage IV in Japan.

This section reports the result of applying the proposed HUSDOS-Boost to original clinical HR data to detect patients with stomach cancer. In addition, possible factors of stomach cancer development estimated by the variable importance of HUSDOS-Boost are discussed.

### 5.1. Health Examination Data

The clinical HR data were collected from the Japanese Red Cross Kyoto Daini Hospital, which provides comprehensive health examination menus. The Research Ethics Committee of the Japanese Red Cross Kyoto Daini Hospital approved the use and analysis of the HR data. Written informed consent was not obtained in this study.

The original HR data were collected between 2014 and 2015, on more than 100 items, including observations, body measurements, blood examination, medical history, and lifestyle. Since some records belonged to the same person collected in both years, we extracted records measured in the year that stomach cancer was initially diagnosed as patient records and the latest records of persons without stomach cancer as healthy records. Persons who had other types of cancer or a prior stomach operation were eliminated from the analysis. The item “gastroscopy result” was not used as an input variable for stomach cancer detection because it is almost identical to the outcome. In addition, the item “family history of stomach cancer” was eliminated. *Helicobacter pylori* is an essential risk factor for stomach cancer development, in which its main infection path is a family member. Only continuous and binary variables were analyzed here because descriptive variables such as “observations” were difficult to analyze.

Finally, the objective data consisted of 7,379 healthy person records (male: 3,890, female: 3,489, age: 56.6 ± 11.6 years old) and 16 patient records (male: 10, female: 6, age: 68.8 ± 10.8 years old); that is, its imbalance ratio was 0.2%. Twelve out of sixteen patients had tubular adenocarcinoma, and the other four patients had either stage IA or IB signet ring cell carcinoma. Forty-one items were adopted as input variables, which are shown in Table 4. “Type” in this table denotes a variable type: a numerical variable (N) and a binary variable (B). No. 1 “Gender” was male/female, and No. 38-41, which asked about lifestyle habits, was yes/no. The data contained about 13% missing values because examination menus vary for each person.

**Table 4:** Input variables.

**No**.	**Description**	**Type**
1	Gender	B
2	Age	N
3	Height	N
4	Weight	N
5	Degree of obesity	N
6	Body fat percentage	N
7	C-reactive protein	N
8	Total protein	N
9	Albumin	N
10	A/G ratio	N
11	Bilirubin	N
12	ALP	N
13	γ GTP	N
14	GOT	N
15	GPT	N
16	LDH	N
17	Cholinesterase	N
18	ZTT	N
19	BUN	N
20	Creatinine	N
21	eGFR	N
22	Uric acid	N
23	Na	N
24	K	N
25	Cl	N
26	Ca	N
27	Cholesterol	N
28	Neutral fat	N
29	HDL cholesterol	N
30	Amylase	N
31	LDL cholesterol	N
32	White blood cell count	N
33	Red blood cell count	N
34	Hemoglobin content	N
35	Hematocrit	N
36	Platelet count	N
37	fasting blood sugar level	N
38	Habit of quick eating	B
39	Habit of meal before sleep	B
40	Habit of breakfast	B
41	Habit of smoking	B

### 5.2. Procedure

The present work applied RF, AdaBoost, SMOTE, ADASYN, RUSBoost, HUSBoost, hyperSMURF, and the proposed HUSDOS-Boost to the HR data for stomach cancer detection. Before analysis, missing values in the HR dataset needed to be input appropriately.

Multiple imputations were used for missing value imputation, which generates multiple complete datasets by replacing missing values with plausible values generated from the posterior distribution of missing values and aggregates them into the final complete dataset (41). We used multiple imputations using chained equations (MICE), which is a standard methodology for coping with HR data with missing values (42). MICE approximates the posterior distribution by regressing it on all other remaining variables. Categorical variables (No. 1 and 38-41) were digitized.

The input data were randomly divided into ten subsets, of which nine were used for modeling while the remaining one was used for validation. Modeling and validation were repeated ten times so that all subsets became the validation dataset once. The above procedure was repeated ten times for precise performance evaluation. The experimental settings of seven methods were the same as section 4.

### 5.3. Results

Table 5 shows the sensitivities, the specificities, the G-means, AUC, and AUPRC in which the bold fonts indicate the best score in the seven algorithms. RF, AdaBoost, and SMOTE did not function because their sensitivities stayed zero while their specificities were almost one. Thus, these algorithms classified all records as healthy. ADASYN improved the classification performance of SMOTE. On the other hand, the performance of hyperSMURF was not improved.

**Table 5:** Stomach cancer detection results.

	**RF**	**AdaBoost**	**SMOTE**	**ADASYN**	**RUSBoost**	**HUSBoost**	**hyperSMURF**	**HUSDOSBoost**
Sensitivity	0.00±0.00	0.00±0.00	0.00±0.00	0.30±0.08	**0.76±0.06**	0.46±0.05	0.14±0.02	0.59±0.07
Specificity	**1.00±0.00**	**1.00±0.00**	**1.00±0.00**	0.93±0.00	0.61±0.01	0.87±0.00	0.98±0.00	0.80±0.00
G-mean	0.00±0.00	0.00±0.00	0.00±0.00	0.53±0.07	0.68±0.02	0.63±0.03	0.36±0.02	**0.69±0.04**
AUC	0.54±0.02	0.62±0.02	0.58±0.02	0.61±0.01	0.56±0.03	0.76±0.00	0.75±0.01	**0.79±0.00**
AUPRC	0.00±0.00	0.01±0.00	0.00±0.00	0.01±0.00	0.00±0.00	0.01±0.00	**0.02±0.01**	0.01±0.00

RUSBoost achieved the highest sensitivity, and HUSDOS-Boost and HUS-Boost were the second and the third best. On the other hand, the specificity of HUSDOS-Boost was higher than RUSBoost. Accordingly, the proposed HUSDOS-Boost achieved the best G-mean and AUC. This result agrees with the result of the case study described in section 4.4. Since the number of patients in the HR data was smaller than 30, the G-mean of HUSDOS-Boost was higher than that of RUSBoost.

**Figure 4:**
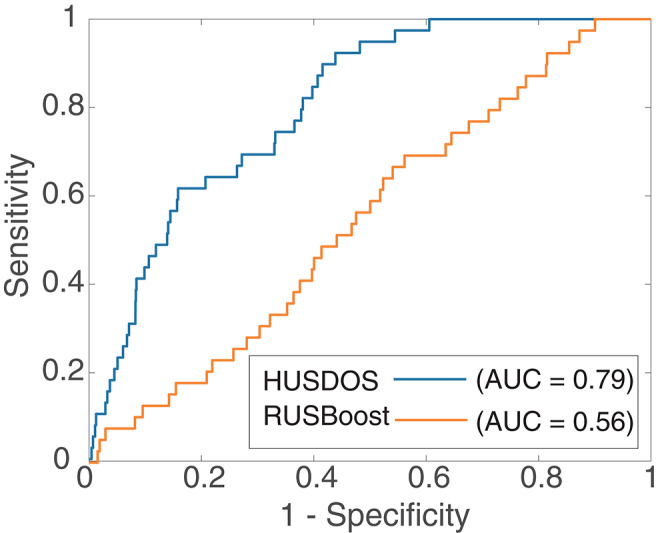
ROC of HUSDOS-Boost and RUSBoost.

**Figure 5:**
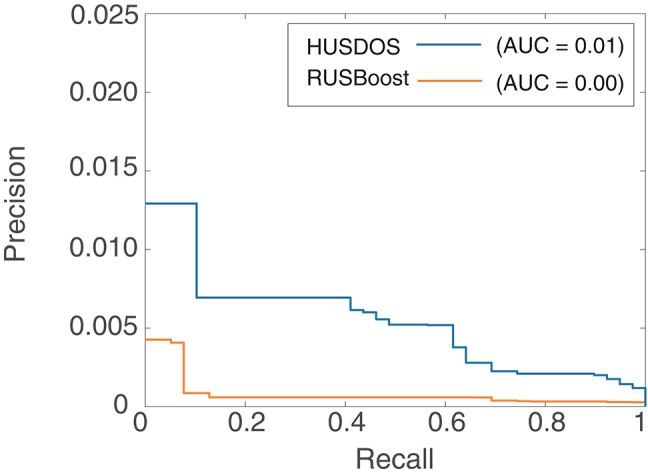
PRC of HUSDOS-Boost and RUSBoost.

AUPRC, however, was almost zero in all algorithms in the HR data. Figures 4, 5 are the ROC and PR curves drawn by RUSBoost and HUSDOS-Boost. Their sensitivity (recall) and specificity were not low, and their precision was close to zero, which indicates that many false positives were detected. In this data, the number of cancer patients was extremely small (0.02%) and consequently the number of true positives became small in comparison with that of false positives. This result suggests that AUPRC is not always appropriate for classification performance evaluation of the EISM data problem.

Although, at the present moment, HUSDOS-Boost cannot be applied to stomach cancer detection using the HR data due to its unsatisfactory performance, the result above suggests the future applicability of the proposed HUSDOS-Boost to patient detection by means of HR data analysis, particularly when the number of patient records in the HR data is extremely small.

### 5.4. Variable Importance

**Figure 6:**
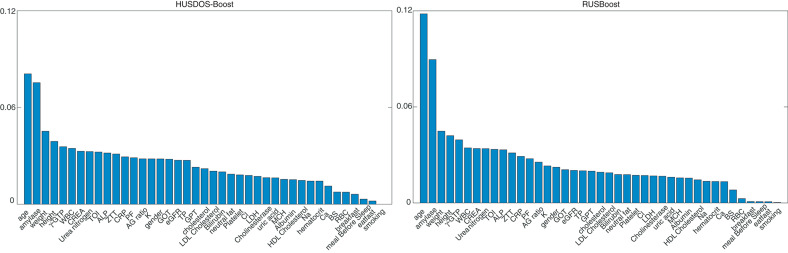
Variable importance: HUSDOS-Boost **(left)** and RUSBoost **(right)**.

The variable importance of stomach cancer detection was calculated using RUSBoost and HUSDOS-Boost, which achieved high G-means. Figure 6 shows the variable importance derived by RUSBoost and HUSDOS-Boost, respectively. “Age” and “amylase” had high importance in both methods.

Age is a factor in stomach cancer development, wherein the morbidity of stomach cancer increases in people over 40 years of age. The mean age of patients was different from healthy persons in the HR data as described in section 5.1. Both methods correctly isolated the factor of stomach cancer from the HR data.

The mean values of amylase were different between patients and healthy persons in the HR data: 88.0 ± 35.8 IU/l of healthy persons and 113.6 ± 45.0 IU/l of patients. They were significantly different (*p* = 0.0075, Effect size: *d* = 0.66, and Power: 1 − β = 0.57); however, the power was rather low due to the sample size of patients being very small. Although salivary gland disorders or pancreatic diseases are suspected when the value of amylase is high, the amylase value becomes high in the elderly population due to the deterioration of amylase clearance in the kidney with age (43). There was the possibility that the values of amylase showed the difference in the mean age between patients and healthy persons. Of course, this result might suggest an unknown relationship between abnormality in amylase and stomach disease, which is difficult to confirm.

Here, we calculated variable importance for another purpose in order to validate the accuracy of the variable importance. Classifiers that detect persons experiencing gastric resection were built by RUSBoost and HUSDOS-Boost, which were utilized for variable importance calculation. Two hundred seven persons experienced gastric resection and did not have stomach cancer at the time of the health examination. The G-means of the classifiers constructed by RUSBoost and HUSDOS-Boost were 0.80 ± 0.01 and 0.77 ± 0.00, respectively. The classification performance of RUSBoost was higher than the proposed HUSDOS-Boost because the number of minority examples, in this case, was more than 40.

Both methods showed that “Age” and “Ca” have the first and the second highest importance for detecting persons with gastric resection. Although there are several causes of persons experiencing gastric resection, they usually occur after middle age. In the HR data, ages of persons with and without gastric resection were 64.9 ± 10.3 and 56.0 ± 11.4, respectively.

In order to confirm the effect of “Age” on the result, we tried to detect stomach cancer without “Age,” whose results are shown in Table 6. The detection performance in every method deteriorated when “Age” was not used. This indicated that “Age” certainly contributed to stomach cancer detection. In addition, the proposed HUSDOS-Boost still achieved the best detection performance.

**Table 6:** Stomach cancer detection results without “Age.”

	**RF**	**AdaBoost**	**SMOTE**	**ADASYN**	**RUSBoost**	**HUSBoost**	**hyperSMURF**	**HUSDOSBoost**
Sensitivity	0.00±0.00	0.00±0.00	0.00±0.00	0.25±0.02	0.72±0.11	0.29±0.06	0.11±0.02	**0.47±0.04**
Specificity	1.00±0.00	1.00±0.00	1.00±0.00	0.94±0.00	0.57±0.01	0.90±0.00	**0.99±0.00**	0.82±0.00
G-mean	0.00±0.00	0.00±0.00	0.00±0.00	0.48±0.02	0.64±0.04	0.51±0.06	0.32±0.03	**0.62±0.03**
AUC	0.54±0.01	0.60±0.02	0.55±0.01	0.59±0.02	0.54±0.03	0.69±0.01	0.70±0.01	**0.71±0.01**
AUPRC	0.00±0.00	0.01±0.00	0.00±0.00	0.00±0.00	0.00±0.00	**0.01±0.00**	**0.01±0.00**	**0.01±0.00**

It is well-known that absorption of Ca decreases after gastric resection (44). The Ca values of persons with gastric resection were lower than persons without gastric resection in the HR data: 9.05 ± 0.31 mg/dL and 8.98 ± 0.33 mg/dL of persons with gastric resection, respectively, which were significantly different (*p* = 0.026, Effect size: *d* = 0.22, and Power: 1−β = 0.88). These results agree with pathological knowledge about the effect of gastric resection. Therefore, this case study shows that variable importance can be applied in the future to the discovery of hidden factors of disease development from HR data.

### 5.5. Limitations

Limitations include properties of the collected data, such as the fact that all records were from a single hospital and that all records were from the Japanese population. Accordingly, more studies using health records collected from other hospitals are required to confirm our results.

## 6. Conclusion and Future Works

The present work proposed a new boosting-based method for handling EISM data by combining HUS and DOS. The case study using eight imbalanced datasets showed that the proposed HUSDOS-Boost achieved comparable performance to RUSBoost when the number of minority examples was more than 40 and that HUSDOS-Boost achieved the best performance when the number of minority examples was smaller than 30. The proposed HUSDOS-Boost was sufficiently fast for learning.

We applied HUSDOS-Boost to the clinical HR data for detecting patients with stomach cancer. The application result showed that the G-mean of HUSDOS-Boost was 0.69. The possible factors of stomach cancer development derived from the variable importance were discussed.

In future works, the hierarchical Bayes model will be introduced to estimate the distribution parameter in DOS in order to improve the over-sampling performance. We will apply the proposed method to clinical HR data to detect other diseases.

## Data Availability Statement

The health examination data will be made available by the corresponding author to colleagues who propose a reasonable scientific request after approval by the institutional review board of the Japanese Red Cross Kyoto Daini Hospital.

## Ethics Statement

The studies involving human participants were reviewed and approved by the institutional review board of the Japanese Red Cross Kyoto Daini Hospital. The ethics committee waived the requirement of written informed consent for participation.

## Author Contributions

KF, YH, and KH and contributed to algorithm development and clinical data analysis, as well as writing the manuscript. KN, MKam, and MKob collected and organized the data analyzed in this study and interpreted the analysis results. MKan managed study implementation, critically reviewed and edited the manuscript, and gave final approval for submission.

## Conflict of Interest

KF is with Quadlytics Inc. as well as Nagoya University. KH and MKan is with Quadlytics Inc. as well as Kyoto University. The remaining authors declare that the research was conducted in the absence of any commercial or financial relationships that could be construed as a potential conflict of interest.
